# A rare case of IgG4-related aortitis in the thoracic aorta mimicking an intramural hematoma: navigating the diagnostic labyrinth

**DOI:** 10.1186/s13019-024-03026-w

**Published:** 2024-10-08

**Authors:** Victor S. Alemany, Jacqueline Fortier, Himanshu Gupta, Arik Zaider, Juan Grau, Paul Burns, Habib Jabagi

**Affiliations:** 1 Division of Cardiothoracic Surgery, The Valley Hospital, 223 N Van Dien Ave, Ridgewood, NJ 07450 USA; 2 Division of Radiology, The Valley Hospital, Ridgewood, NJ 07450 USA; 3 Division of Rheumatology, The Valley Hospital, Ridgewood, NJ 07450 USA; 4 Division of Cardiac Surgery, University of Ottawa Heart Institute, Ottawa, ON K1Y 4W7 Canada; 5 Department of Cardiovascular Surgery, Mt. Sinai Hospital, Icahn School of Medicine, New York, NY 10029-6574 USA

**Keywords:** Aortitis, IgG4-RD, Intramural hematoma, Acute aortic syndrome, Aortic imaging, Ascending aorta

## Abstract

A 54-year-old female presented with recurrent abdominal pain and new onset chest pain. Chest computed-tomography angiogram detected a thoracic aortic aneurysm with *suspected* Type A intramural hematoma (IMH) versus aortitis. Initially, conservative management was pursued while awaiting a definitive diagnosis. Differential workup was negative, while additional imaging modalities favored IMH, prompting expedited surgical intervention. During ascending aortic and hemiarch replacement, severe aortitis was unexpectedly discovered without evidence of IMH. Histopathological examination of the aortic specimens identified transmural aortic inflammation with lymphoplasmacytic infiltrate and irregular fibrosis. Numerous IgG4-positive plasma cells were present with IgG4/IgG ratio of 40–50% suggesting IgG4-related disease (IgG4-RD). Subsequent analysis revealed B cells positive for clonal IgH gene rearrangement, and bone marrow biopsy then revealed the same clonal B cells. She was ultimately diagnosed with CLL, the most common phenotype of monoclonal B-cell lymphocytosis, thought to account for the IgG4-predominant plasma cells causing aortitis. Although rare, this case highlights the importance of considering IgG4-related disease (IgG4-RD) as a cause of aortitis when assessing symptomatic patients with aortic pathologies, emphasizing the complexities involved in diagnosing due to a variety of imaging presentation, differentiating, and managing large-vessel vasculitides. Moreover, it underscores the importance of Multidisciplinary Aortic Team care and the use of multiple diagnostic modalities in evaluating ambiguous aortic pathologies.

## Case summary

A 54 year-old female presented with new onset chest pain radiating to her upper back, associated with recurrent abdominal pain, general malaise, and a 3-days history of nausea and vomiting. Notably, she was hospitalized 2-months ago for abdominal pain, and diagnosed with acute bilateral renal infarcts (left > right) of unknown etiology. Other comorbidities included: recent COVID-19 infection, bronchitis, beta-thalassemia, migraines, temporomandibular joint dysfunction, inflammatory arthritis consistent with psoriatic arthritis. Her past medical history was otherwise significant for previously elevated erythrocyte sedimentation rate (ESR) and C-reactive protein (CRP) levels, oral corticosteroid use (> 1 year ago), and occasional corticosteroid injections for shoulder pain. Her family history was negative for any aneurysms, dissections, early or sudden death, and connective tissue diseases. The patient is a lifelong non-smoker, and had no relevant past surgical history, recent travel, allergies, history of infectious diseases, including syphilis. Review of systems was positive for headache, jaw pain, rhinitis, and fatigue. There was no history of salivary/lacrimal gland enlargement, dry eyes, lymphadenopathy, limb claudication, pancreatitis, cholangitis, oral or genital ulcers, rash, fevers, night sweats, or weight loss.

On physical exam, the patient was comfortable with a heart rate of 83 bpm, and a blood pressure of 150/65 mmHg, with symmetrical/palpable radial, femoral, and pedal pulses. She had normal S1 and S2 heart sounds, with no murmurs or signs of heart failure. No lymph node or salivary gland enlargement. No abdominal pain or swelling. Hematological workup was negative for any hypercoagulable disorders or malignancies with a normal white blood cell count (WBC) and differential, negative D-dimer and troponins, and normal creatinine. Electrocardiogram (ECG) showed normal sinus rhythm with no ST changes concerning for ischemia. Transesophageal echocardiogram (TEE) from previous admission was normal with no intracardiac thrombus or atrial septal defect (ASD) identified. A chest computed-tomography angiogram (CTA) revealed a thoracic aortic aneurysm (TAA) affecting the ascending aorta (maximum 5.3 cm), with abnormal nonspecific circumferential diffuse wall thickening extending from the root to the aortic arch—highly concerning for intramural hematoma (IMH). Radiology also reported the possibility of an associated inflammatory aortitis, and no evidence of aortic dissection. Due to the location of her aortic pathology and presenting symptoms, a diagnosis of acute aortic syndrome (AAS) was considered along with noninfectious aortitis (large-vessel vasculitides). Of the three AAS, IMH was initially suspected based on CTA findings. Other differentials considered included: enlarging, symptomatic TAA, contained aortic rupture, penetrating aortic ulcer, and infectious aortitis.

**Fig. 1:**
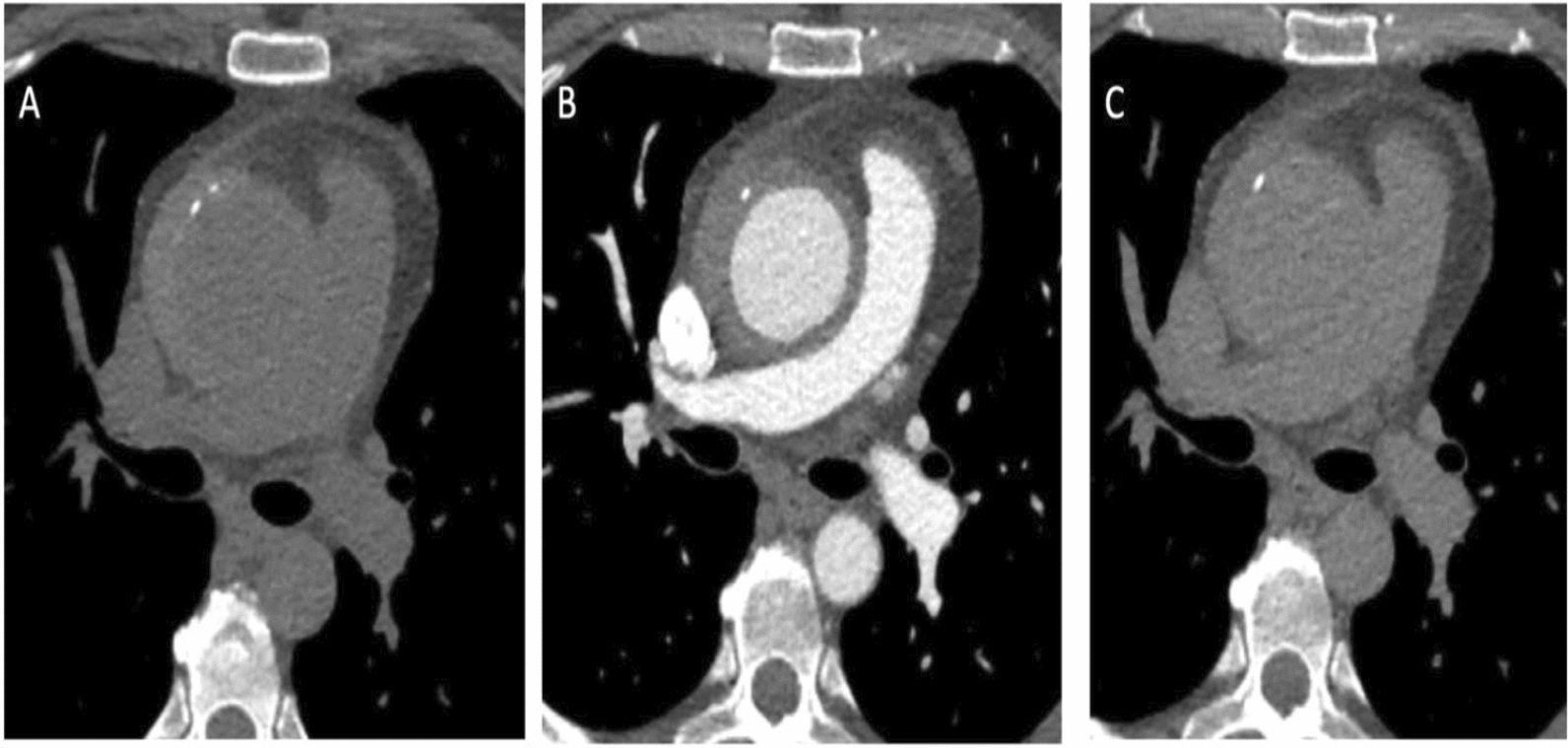
Preoperative ECG gated axial CTA images of the ascending aorta. **A** Pre-contrast, **B** Contrast, **C** Post-contrast. Crescent-shaped, high attenuation, non-enhancing wall thickening of the ascending aorta, beginning at the origin of the right coronary artery and terminating prior to the takeoff of the innominate artery. Favored to reflect Type A aortic intramural hematoma, due to the absence of enhancement, high density thickening (60 Hounsfield units), and maximum thickness (13 mm). Ascending aorta measured 5.3 cm in diameter with crescent and 3.8 cm without. Several calcifications between the lumen and the external aortic wall were also noted circumferentially. Abbreviations: *CTA* computed-tomography angiogram, *ECG* electrocardiogram

**Fig. 2:**
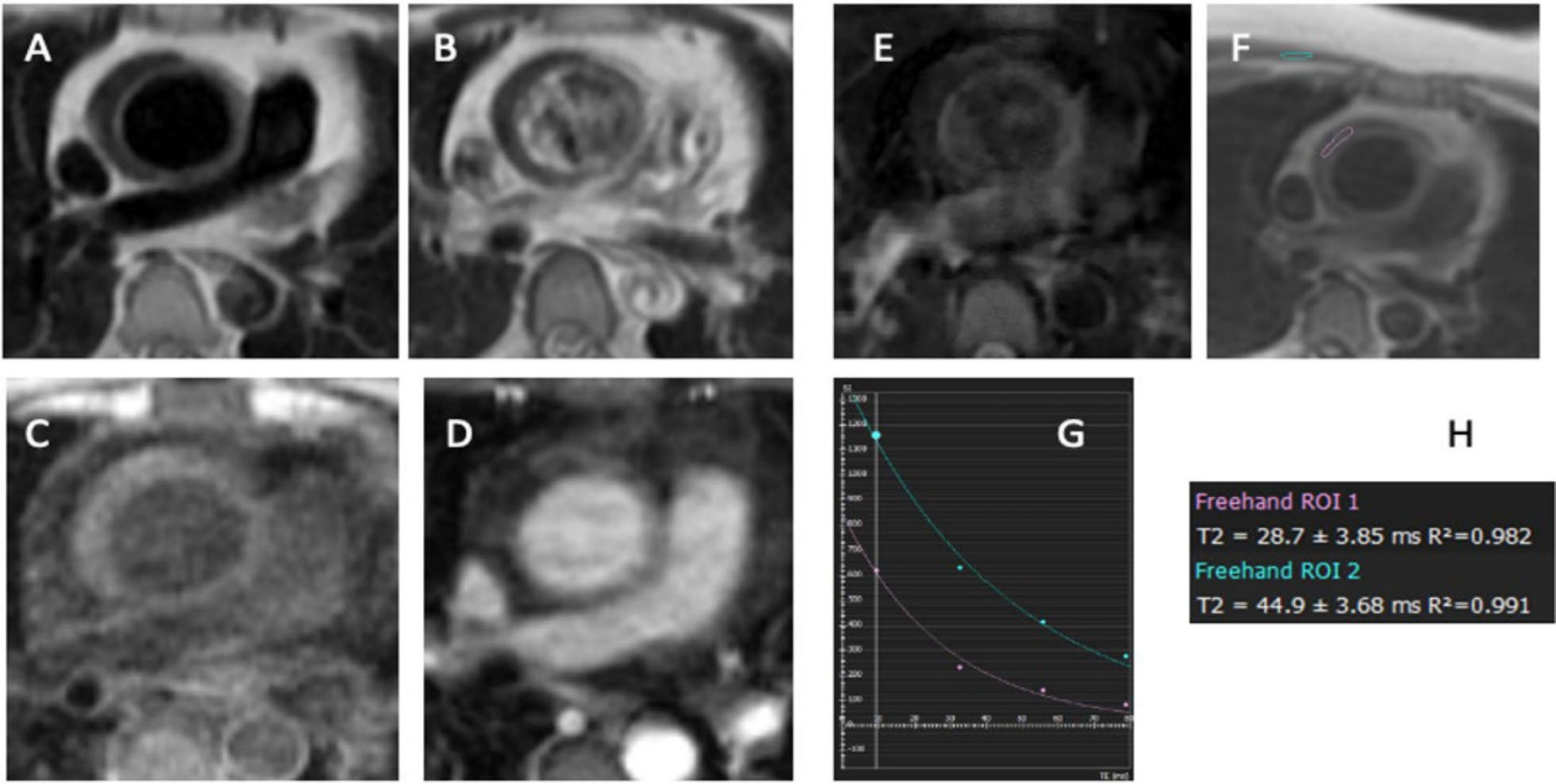
Preoperative ECG gated axial MRI images of the ascending aorta. ECG gated MRI of the ascending aorta: **A** Pre-contrast T1 weighted spin echo image; **B** Post-contrast T1 weighted spin echo image; **C** Pre-contrast LAVA image; **D** Post-contrast LAVA image; **E** T2 weighted fat saturation spin echo image; **F**–**H** T2 mapping of the aortic wall compared to skeletal muscle. T1 weighted pre-contrast image (**A**) demonstrates a crescent-shaped low signal intensity area within the aortic wall which does not change post-contrast (**B**). Similarly, the pre-contrast LAVA image (**C**) does not demonstrate enhancement post-contrast (**D**). T2 images (**E**) demonstrate hypointense signal intensity in the aortic wall, which on T2 mapping demonstrates a lower T2 value compared to skeletal muscle (**G** and **H**). Together, these findings are supportive of aortic wall intramural hematoma rather than aortitis. Abbreviations: *ECG* electrocardiogram, *LAVA* liver acceleration volume acquisition, *MRI* magnetic resonance imaging

**Fig. 3:**
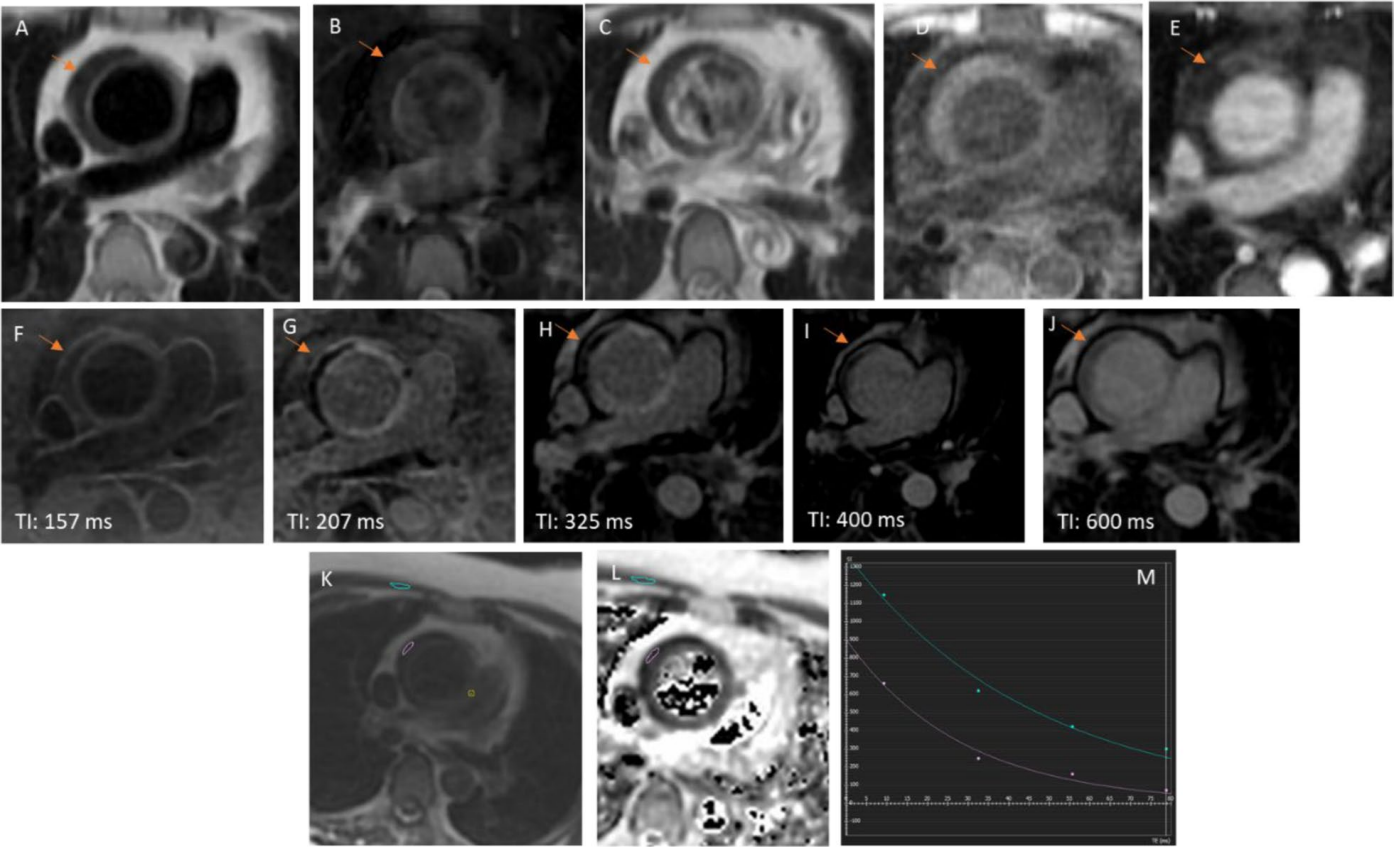
Late Gd-enhancement images (LGE). **A** Pre-contrast T1 weighted spin echo image; **B** Pre-contrast T2 weighted spin echo image; **C** Post-contrast T1 weighted spin echo image; **D** Pre-contrast LAVA image; **E** Post-contrast LAVA image; **F**–**J** Phase sensitive late Gd-Enhancement images; **K**–**M** T2 mapping of the aortic wall compared to skeletal muscle. T1 and T2 weighted pre-contrast image (**A**, **B**) demonstrates a crescent-shaped low signal intensity area (arrow) within the aortic wall which does not change on T1 weighted image post-contrast (**C**). Similarly, the pre-contrast LAVA image (**D**) does not demonstrate enhancement post-contrast (**E**). On late Gd-enhancement images (LGE) performed using Phase-Sensitive Inversion Recovery Sequence (PSIR), the crescentic shaped low signal intensity (arrow) in the aortic wall persisted across varying inversion time (TI). Enhancement of the endoluminal surface of the thoracic aorta adjacent to the crescentic shaped low signal intensity and adjacent aortic wall was present, best characterized on inversion time of 207 and 325 ms. T2 mapping demonstrates a lower T2 value compared to skeletal muscle (**K**–**M**). Together, these findings are supportive of aortic wall intramural hematoma. Associated inflammation of aortic wall is suspected due to presence of LGE of adjacent aortic wall segments

**Fig. 4:**
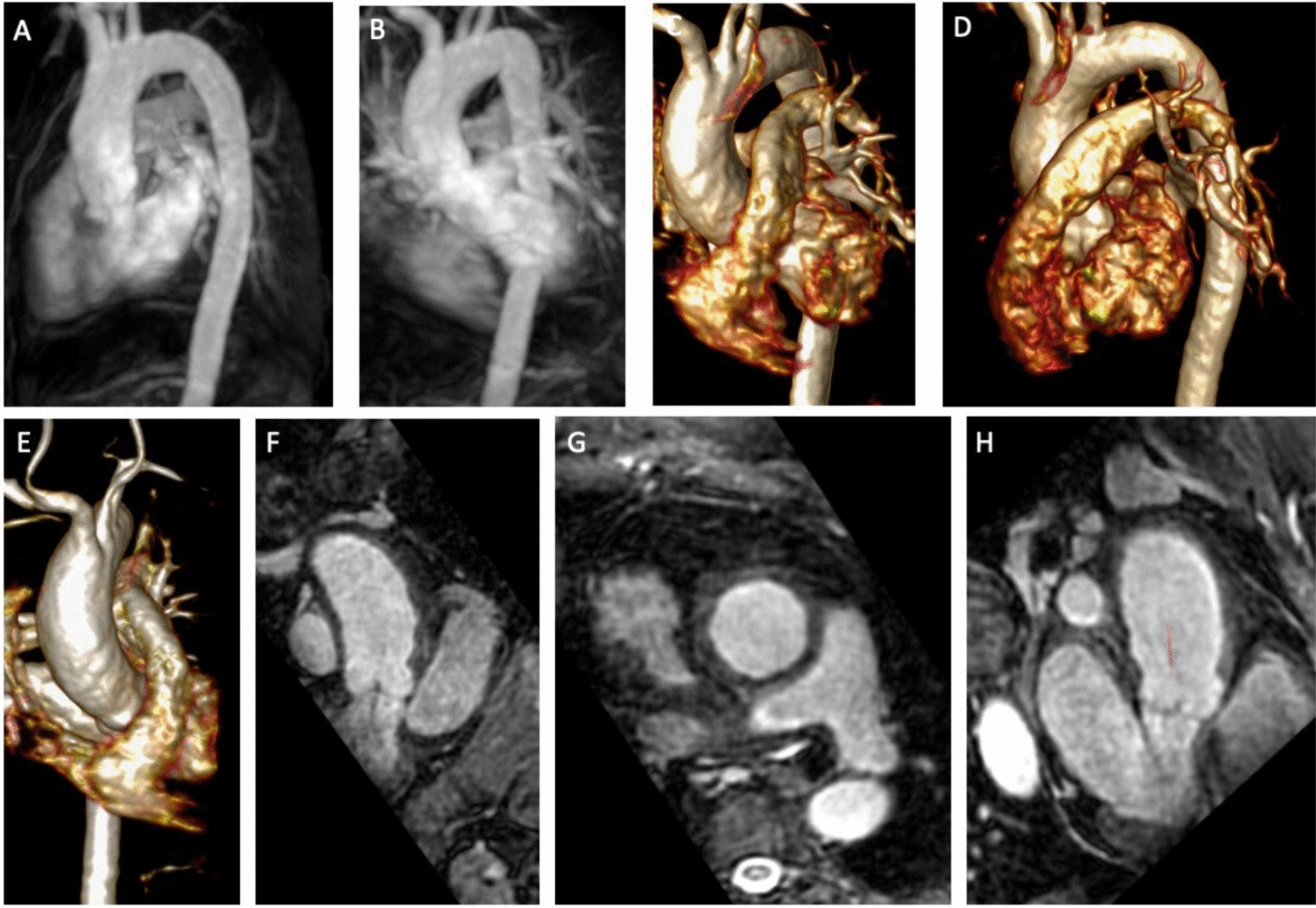
Preoperative multidirectional images of CTA and MRI. **A**, **B** Preoperative 3D contrast enhanced MIP; **C**–**E** 3D contrast enhanced mRA volume rendering; **F**–**H** 3D non contrast MRA

Given the patient's stable hemodynamic status and need for further diagnostic clarification, a conservative management approach was initiated by cardiac surgery. The patient was admitted for further work-up and surveillance, with repeat imaging planned in 7 days, aggressive BP control, and a rheumatology consult. Subsequent labs were significant for elevated ESR (80 mm/h) and CRP (18 mg/L) levels, with negative blood cultures and syphilis serology. Extensive serology panels and inflammatory markers ordered by rheumatology for vasculitis were non-contributory, with only mild, nonspecific elevations of IgG3, Beta-2 IgM, and Phospholipid IgG. The remaining tests were normal including: eosinophil count, HLA-B27, ANA, ANCA, and IgG4. Genetic testing revealed a variance of unknown significance in the MYH 11 gene (heterozygous). A repeat ECG-gated chest CTA on day 7 revealed no significant changes, however improved resolution from ECG-gating enabled a more detailed assessment of the aortic wall; now described as a high density (60 Hounsfield units), eccentrically thickened (maximum 13 mm), crescent-shaped (right lateral aspect) ascending aortic wall, concerning for IMH (Fig. 1). Additional assessment with magnetic resonance angiography (MRA) was performed, revealing additional findings supporting a diagnosis of IMH rather than aortitis (Figs. 2, 3, 4).

In the absence of robust clinical data, the approach to managing IMH relies predominantly on the specific segment of aorta affected—the key determinant of lethality risk (Type A > Type B IMH) [2, 14]. As per the 2022 ACC/AHA Guidelines on the Diagnosis and Management of Aortic Disease, prompt surgical repair is strongly recommended (Class I,B-NR) for patients with acute (complicated and uncomplicated) Type A IMH [18]. However, the decision to proceed with surgery should be individualized. Accordingly, to determine the most appropriate course of action for each patient, patient-specific comorbidities and mortality risks associated with surgical repair are weighed against the potential risk of aortic–related complications with medical management [18]. To assist in this evaluation, several high–risk imaging features have been identified that predict poor outcomes with medical management of Type A IMH, including: maximum aortic diameter > 45–50 mm [22], hematoma thickness greater ≥ 10 mm [17], focal intimal disruption with ulcer–like projection involving the ascending aorta or arch, and presence of pericardial effusion on admission [22, 27].

Given our patient’s symptoms of chest pain with no other attributable cause, the specific location of her aortopathy, and findings of IMH on both CTA and MRA, a decision to proceed with surgical intervention for Type A IMH was made for morbidity and mortality benefit. The patient was a low-risk surgical candidate for open repair, and also exhibited two high-risk IMH image findings, with maximum aortic diameter of 53 mm and hematoma thickness of 13 mm. The patient consented and underwent uncomplicated ascending aortic and hemiarch replacement (using a 24 mm Hemashield Platinum woven graft) with concomitant left atrial appendage ligation.

**Fig. 5:**
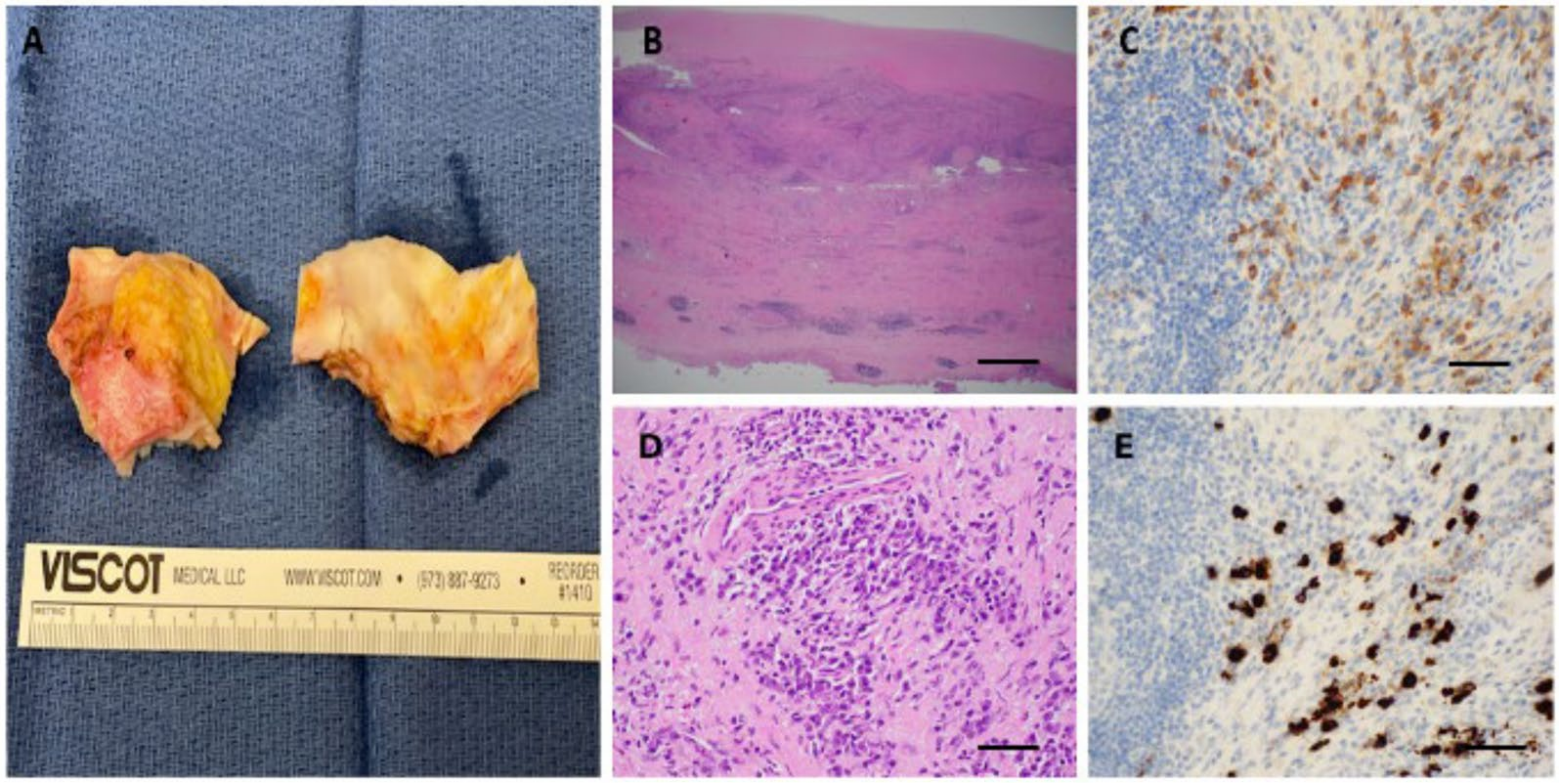
Histopathology ascending aortic specimens. (**A**) Ascending Aorta (Gross Specimen)—diffuse aortic wall thickening with irregular fibrosis, ulceration, and nondescript reparative changes, consistent with chronic active aortitis. **B**, **D** Haematoxylin and Eosin Staining (20 × top, 400 × bottom)—transmural aortic wall inflammation comprised predominantly of lymphocytic infiltrate (top), fibrosis, obstructive phlebitis and eosinophin infiltration, with nearly equal mixture of CD3/CD43 positive T lymphocytes and CD20 positive B lymphocytes along with CD138, lambda and kappa positive polyclonal plasma cells (bottom). These findings are consistent with low-grade B-cell lymphoma and IgG4-related disease in the absence of findings pointing to other autoimmune disease or infection etiology. **B** Scale bar = 500 mm. **D** Scale bar = 100 mm. **C, E** Immunoperoxidase staining (400 ×)—immunohistochemical staining highlights many IgG positive plasma B-cells (top) with IgG4 immunohistochemical staining showing an increased proportion of IgG4-positive plasma cells (bottom). Plasma cell ratios strongly supported a diagnosis of IgG4-RD, with an IgG4 +: IgG + ratio of 40–50%. Scale bars = 100 mm

**Fig. 6:**
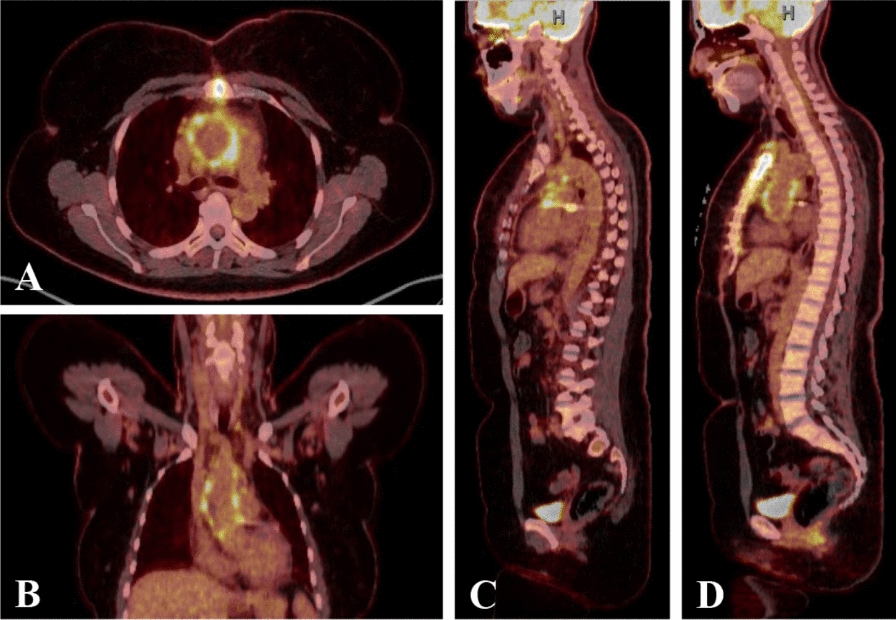
Postoperative FDG-PET CT of the chest. **A**, **B** Hemashield platinum graft replacement of ascending aorta, with circumferential intense activity (SUVmax = 13.7) consistent with graft material. **B**, **C** Sagittal full body views showing the absence of aortic inflammation throughout the remaining portions of the aorta down to the aortic bifurcation. Abbreviations: *CT* computed-tomography, *FDG* fluorodeoxyglucose, *PET* positron emission tomography, *SUV* standardized uptake value

Intraoperatively, the ascending aorta displayed features of severe “burned out” aortitis, with severely fibrotic and thickened aortic tissue, and multiple areas of intramural calcification. (Fig. 5A) Histologically, the aortic wall demonstrated transmural inflammation (intima to adventitia) with a predominantly lymphoplasmacytic infiltration of the adventitia, admixed with neutrophils and histiocytes in a background of irregular fibrosis. Numerous IgG4-positive plasma cells were present with IgG4/IgG ratio of 40–50% suggestive of IgG4-related disease (IgG4-RD) (> 40% is suggestive of IgG4-RD). Subsequent analysis revealed B cells positive for clonal IgH gene rearrangement suspicious for CLL. These findings prompted further testing with targeted imaging for the presence of any vascular inflammation or lymphoproliferative disorders, however none were identified by fluorodeoxyglucose (FDG) positron emission tomography (PET)-CT of the chest. (Fig. 6) In order to confirm the diagnosis, a peripheral blood flow cytometry study was performed which showed a similar B cell clone found in the pathological sample from the Aorta and a bone marrow biopsy that again confirmed the presence of the same clonal immunoglobulin gene rearrangement and clonal B cells that had the same immunophenotype profile (CD5 +, Cd23 +, FMC7 +) of the cells found in the aorta and peripheral blood.

The post-surgery course was free of complications and the patient was discharged in good clinical condition with follow-up referrals to Rheumatology and Oncology. Subsequent bone marrow biopsy and peripheral blood smear as an outpatient again identified clonal B-cells with a chronic lymphocytic leukemia (CLL)—like immunophenotype, identical to those found in the aortic specimens. She was ultimately diagnosed with monoclonal B-cell lymphocytosis(MBL), of which CLL is the most common phenotype, thought to account for the IgG4-predominant plasma cells causing aortitis. Rituximab treatment(375 mg/m^2^ weekly for 4 weeks) was promptly initiated—a recognized treatment of both CLL and IgG4-RD [19, 40]. This resulted in clearance of her peripheral blood flow cytometry and she has been clinically stable since.

## Discussion

Herein we present a rare case of IgG4 aortitis, whose unusual presentation and imaging characteristics not only hindered accurate diagnosis and influenced treatment decisions, but also resulted in the detection of an underlying malignancy, enabling the patient to receive prompt initiation of treatment. Despite collaborative efforts from a multidisciplinary clinical team, comprehensive laboratory tests, and various imaging investigations, establishing a definitive diagnosis in this patient was unattainable without surgery. Intramural hematoma of the aorta characterizes by the accumulation of blood within the aortic wall, typically due to rupture of the vasa vasorum or intimal tear without full-thickness aortic wall disruption. Patients often present with sudden onset severe chest or back pain, similar to aortic dissection, but without the typical pulse deficits or blood pressure differentials seen in dissection, like it happened in this patient. Diagnosis relies heavily on imaging studies, particularly CT angiography, which can visualize the hematoma within the aortic wall.

IMH, the presumed diagnosis, was only ruled out at the time of surgery with direct visualization of the aorta, while histopathology of aortic specimens established the definitive diagnosis of IgG4 aortitis, and later the discovery of an undiagnosed CLL.

Aortitis is a type of vasculitis characterized by inflammation of the aorta and/or its major branches. The most common cause of aortitis in adults is typically large-vessel vasculitides (LVV), which include giant cell arteritis (GCA) and Takayasu arteritis (TAK). These conditions predominantly affect the proximal thoracic aorta, and are relatively uncommon [10, 26, 32, 36]. Due to its complex phenotypic spectrum, LVV frequently present with features and symptoms similar to other diseases of the proximal aorta, including intramural wall hematoma, making accurate diagnoses and subsequent management difficult. Less frequently, aortitis may also occur in association with other forms of vasculitis or underlying conditions, including: inflammatory and rheumatological diseases (i.e. rheumatoid arthritis, Behcet's disease, IgG4-related disease, RA, and SLE), as well as various infections (i.e. syphilis, salmonella, HIV, and tuberculosis) [12].

Typically presenting with nonspecific symptoms such as chest discomfort, fever, and weakness, LVV patients frequently evade diagnosis despite multiple encounters with healthcare providers [36]. This is even more apparent in cases of aortitis caused by IgG4, with case reports in literature highlighting the difficulties in diagnosing this uncommon and under recognized cause of aortitis [36, 39, 41].

Distinguishing chronic aortitis compared to atherosclerosis may be challenging on MRI, as there can be significant overlap between chronic inflammatory changes of aortitis and atherosclerosis. Unique aspects of MRI included comprehensive T2 mapping of aortitis along with LGE performed using different inversion times. These methodologies are complementary to 3D MRA and conventional evaluation of edema and enhancement on MRI [11]. Comprehensive characterization of vascular inflammation using these techniques needs prospective evaluation as currently use of these techniques is not well described in the literature (Fig. 6) [5, 16].

Lacking a definitive diagnostic test and no validated diagnostic criteria, aortitis is usually diagnosed histopathologically after surgery for aortic aneurysms or presumed aortic dissections [8, 12]. No other organ biopsies apart from the bone marrow biopsy were perrfomed to allow for a preoperative diagnosis of IgG4 aortitis, however the patient had a negative MRI of the temporal lobe, which has been found to be a good subrogate for GCA allowing to avoid invasive biopsies.

IgG4 related disease is an immune-mediate fibroinflammatory condition capable of affecting multiple organs. Clinical manifestations may include salivary gland involvement, lymphadenopathy, orbital inflammation, autoimmune pancreatitis, sclerosing cholangitis, retroperitoneal fibrosis, and vascular involvement [20]. The diagnosis of IgG4-RD requires clinical or radiologic evidence of organ involvement and a biopsy that demonstrates lymphoplasmacytic infiltrate with multiple IgG4-positive plasma cells, storiform fibrosis, and obliterative phlebitis. Serum IgG4 is elevated in 2/3 of patients. It is estimated that IgG4-related aortitis occurs in approximately 8 percent of patients with IgG4-RD and accounts for 2–9 percent of all patients with noninfectious aortitis. Although some genetic associations like HLA class II genetic region, genes encoding for cytokines like TNF and their receptors, VEGF and ICAM-1 have been described, there is no particular symptomatology or radiological finding that can help distinguish IgG4 vasculitis from the rest of the large vessel vasculitis spectrum until the histopatological diagnosis is performed [30, 36, 43].

This case exemplifies the critical role of multidisciplinary collaborations when navigating complex aortic conditions such as aortitis. Our patient benefited from a comprehensive treatment team comprising cardiac surgeons, imagining specialists with expertise in aortic disease, a cardiac surgery intensive care unit (ICU) experienced in managing acute aortic diseases, and a rheumatologist, all actively involved in the diagnostic and decision making process. Even with this comprehensive support, uncertainties persisted regarding the presumed definitive diagnosis of IMH in this patient, posing further challenges in determining an appropriate treatment strategy.

Regarding treatment, IgG4-related vasculitis can often be treated medically with corticosteroids and immunosuppressive agents, leaving surgical excision for very severe cases. In contrast, intramural hematoma, when developed, can be managed medically with strict blood pressure control and close monitoring if non-expanding, absence of pain and no complication such as rupture develop, otherwise, surgical intervention is warranted (Table 1).

**Table 1:** Differences between Aortitis and IMH

	Aortitis	IMH
Etiology	Large-vessel vasculitis (giant cell arteritis (GCA) and Takayasu arteritis (TAK)	Rupture of the vasa vasorum or intimal tear
Pathology	Inflammation of the aorta and/or its major branches	Accumulation of blood within the aortic wall
Symptomatology	Chest discomfort, fever, and weakness	Sudden onset severe chest or back pain
Diagnosis	3D MRA, Late gadolinium enhancement (LGE) MRI Definitive: histopathological	CT angiography shows hematoma on Aortic wall
Treatment	Medically with corticosteroids and immunosuppressive agents, Very severe cases: surgical excision	Medically with strict BP control Expanding or symptomatic: surgery

Given most data available on type A IMH favors surgical intervention, with IRAD reporting mortality rates of 40% with nonoperative strategies, surgical intervention was heavily favored in our patient [7, 15, 17, 29, 39]. Taking into account the patient’s characteristics, low surgical risk, and high risk IMH image findings, surgical intervention was established as the treatment of choice. Alternatively, had a diagnosis of aortitis been recognized, the patient may have been able to forego surgery and receive treatment directed at IgG4-RD. However, this approach would have left diseased and weakened aorta behind in a young patient, placing her at an increased lifelong risk of suffering a potentially lethal adverse aortic event. Irrespective of etiology, aortitis is related to significant morbidity and mortality, due to the development of complications such as aortic aneurysm, aortic rupture, aortic dissection, and/or thrombotic luminal obstruction [8, 12]. Particularly, aortitis of the ascending aorta is known to be associated with ascending aortic dissection and increased mortality [23, 41].

Another intriguing aspect of this case and rare diagnosis of IgG4-related aortitis, is its potential association with malignancy. Research indicates a potential precursor connection between IgG4-related conditions and invasive malignancies, such as prostate cancers, lymphomas, myeloproliferative neoplasm and acute myeloid leukemia [41, 42]. Interestingly, our patient was later diagnosed with concurrent chronic lymphocytic leukemia (CLL); however, determining which condition preceded the other remains uncertain. Fortunately, both conditions share the same treatment approach, offering some solace for the patient.

## Conclusion

IMH and Aortitis are very different pathological processes with very different treatment approaches that can present with very similar symptomatology and imaging characteristics. A multimodality diagnostic approach and pathological study of tissue obtained during surgical intervention are sometimes necessary for the obtention of a definitive diagnosis and appropriate treatment strategy.

## Data Availability

No datasets were generated or analysed during the current study.
